# Futile Recanalization after Endovascular Therapy in Acute Ischemic Stroke

**DOI:** 10.1155/2018/5879548

**Published:** 2018-05-09

**Authors:** Ximing Nie, Yuehua Pu, Zhe Zhang, Xin Liu, Wanying Duan, Liping Liu

**Affiliations:** ^1^Department of Neurology, Beijing Tiantan Hospital, Capital Medical University, Beijing, China; ^2^China National Clinical Research Center for Neurological Diseases, Beijing, China; ^3^Center of Stroke, Beijing Institute for Brain Disorders, Beijing, China

## Abstract

Early recanalization after endovascular treatment could improve the prognosis of acute ischemia stroke. Futile recanalization often occurred which was one of the main causes of failure. By now the mechanisms of futile recanalization were not clear. They are probably concerned with bad collateral circulation, subacute reocclusion, large hypoperfusion volumes, microvascular compromise, and impaired cerebral autoregulation. Previous research found that some of the image markers could be used as the accurate predictors for poor prognosis after successful treatment in order to identify the patients who were not suitable for recanalization and reduce some of the unnecessary cost. Predictors for futile recanalization mentioned in our article can be used for supplement to make decision for endovascular treatment.

## 1. Introduction

It has been proved that early recanalization could reduce mortality of acute ischemic stroke patients with large vessel occlusion and improved the prognosis finally. Five randomized controlled trials proved the efficacy of endovascular treatment over standard medical care in patients with acute ischemic stroke caused by large vessel occlusion of the proximal anterior circulation [1–4]. For many interventional radiologists, the main goal of endovascular treatment is to complete recanalization. However, it was upsetting sometimes that not all patients had good clinical outcome at the end despite a perfect revascularization. It often occurs when successful recanalization fails to bring favorable prognosis which called futile recanalization. Studies with first-generation IA thrombectomy device found that the rate of futile recanalization after endovascular treatment was over 60% which had not improved outcomes compared with intravenous thrombolysis alone [5]. It was still a challenge when determination was made by neurologist or interventional radiologist after a great success of second-generation IA thrombectomy devices. A meta-analysis of individual patient data from the trials with the MR CLEAN, EXTEND-IA, ESCAPE, SWIFT PRIME, and REVASCAT found that the rate of futile recanalization after endovascular treatment was 54% [6]. In this review, we will highlight the predictors for poor outcome following endovascular treatment.

We performed a major online database search including Medline, PubMed, Cochrane Library, and EMBASE to identify any related articles using the key words “futile recanalization”. There was not a clear definition of futile recanalization. Some of pervious papers summarized it as poor clinical outcomes after an adequate vessel recanalization for patients with acute stroke [7]. In another study, “futile recanalization” was defined by the occurrence of poor functional outcome (mRS score larger than 3 at 1–3 months) despite complete angiographic recanalization (TIMI grade 2b or 3) [5].

## 2. Causes and Mechanism for Futile Recanalization

### 2.1. Futile Recanalization and Cerebral Blood Flow Regulation

Previous research had studied the correlation between cerebral blood flow regulation and outcomes after using recombinant tissue plasminogen activator (rtPA) for acute cerebral infarction [8]. Previous publications on cerebral autoregulation in ischemic stroke found a transient impairment of cerebral autoregulation during the subacute stages of sever cerebral infarction [9–11]. Surprisingly, animal study showed that rtPA displays neurotoxic properties. According to the results of this study, the use of rtPA might destroy the blood-cerebral barrier, damage blood vessels, and impair the cerebral autoregulation possibly [12]. But the outcome of humans study was opposite which concluded that the use of rtPA did not conduce to impaired cerebral autoregulation. However, cerebral autoregulation after rtPA treatment in this study was evaluated 10–20 h later, so it was possible to ignore the initial detrimental effect by rtPA on cerebral autoregulation [8]. Some researchers believe that impaired cerebral autoregulation may be one of the possible mechanisms of futile recanalization. The mechanisms of cerebral blood flow regulation during and after intravenous thrombolysis are not clear, not to mention the mechanical recanalization. More research focused on the evaluation of cerebral autoregulation influenced by endovascular treatment is needed to explore the correlation with futile recanalization.

### 2.2. Futile Recanalization and Hypoperfusion Volume

The existence and the range of ischemic penumbra which is salvageable potentially can conceivably change the prognosis. Hypoperfusion volumes could indicate final infarct volume [13]. It was observed in over 40% of patients after intra-arterial thrombolysis recanalization a few hours later [5]. It has been demonstrated that most hypoperfused tissue developed into real infarction in seven days. Initial hypoperfusion was linked to selective neuronal loss in rescued penumbra probably [14]. The patients with large hypoperfusion volumes were more likely to have poor prognosis.

### 2.3. Futile Recanalization and Collateral

The prognosis could be influenced by collateral circulation which made tissue viability sustain until effective recanalization [15]. Proximal occlusion usually affected larger areas of brain tissue and often came to poor outcome finally [16]. Reocclusion after recanalization often occurs immediately [17], hours later, or in the first 24 hours. Previous research found that reocclusion was associated with neurologic deterioration which occurred in nearly 10% of patients receiving intra-arterial thrombolysis [18–20].

### 2.4. Futile Recanalization and Microvascular Compromise

Another important factor is microvascular compromise which could influence effective tissue perfusion despite macrovascular recanalization at the capillary level. It occurs after leukocytes and platelets aggregation causing plugging of microvessels due to endothelial activation. These changes of microcirculation disturbance have been associated with unfavourable prognosis following percutaneous transluminal coronary angioplasty (PTCA) after the first attack of AMI [21]. This issue about acute stroke has not been studied up to now.

### 2.5. Technology Difference between IA Thrombectomy Devices

As we mentioned before, the use of the second-generation IA thrombectomy device such as Solitaire improved the prognosis compared with the first-generation IA thrombectomy device such as Merci. The occurrence rate for FR was a little decreased. It is believed that technical progress of method of endovascular treatment may bring about new changes. But until now the occurrence rate for FR was still high, although with these new thrombectomy device we could improve the prognosis finally. And most of the time the mechanism for occurrence of futile recanalization was similar. The exact physiopathologic mechanisms of different thrombectomy device are unclarified yet.

By now the mechanisms of futile recanalization were not clear. They are probably concerned with bad collateral circulation, subacute reocclusion, large hypoperfusion volumes, microvascular compromise, and impaired cerebral autoregulation.

## 3. Prediction for Futile Recanalization

Severe cerebrovascular disease not only brings body's illness and the life pressure to patients, but also brings serious economic burden to patients' family. The aim of reperfusion therapy in acute ischemia stroke as we expect is not only to recanalize the occluded vessel but also to save the ischemic but still viable brain tissue. It could promote the prognosis of most patients with acute stroke which is the most effective treatment at present. However, these treatments are often expensive and are often not available on Social Security and Medicare. Some of the families can hardly afford the cost of such bridging treatment. The reliable prediction of futile recanalization is important which can identify the patients who are not suitable for recanalization and reduce some of the unnecessary cost.

### 3.1. Clinical Features

In a multicenter study [5], individual data of acute ischemic stroke treated with endovascular treatment combined from six studies were analyzed. 96 of 270 patients after intra-arterial thrombolysis achieved complete recanalization. It had been observed that 47 patients (49%) satisfied the definition of futile recanalization. High baseline NIHSS score (NIHSS score > 10), older age (age > 70 years), and longer delay have been identified as possible predictors for poor outcome after complete recanalization [22]. This is the first article focused on futile recanalization, especially among elderly and severe patients. These findings had been confirmed by following studies. But the sensitivity of these factors mentioned was not high enough. And the data of six studies had obvious heterogeneity and some of them did not use the new technique of endovascular therapy. Reliable makers need to be established.

Some clinical features could be used for early discrimination for futile recanalization. High baseline NIHSS score was associated with FR, which was mentioned in most of the research about FR, although some research found that the patients with high NIHSS score could be benefit from endovascular treatment. But there is no avoiding the fact that high baseline NIHSS score is more likely for the occurrence of FR [23–25]. It was a controversial issue about age and many doctors hold quite different opinions. Available data suggested that patients with older age easily developed FR [5, 25]. Another clinical features mentioned more frequently included longer delay and ischemic lesion which might be associated with FR in some research. Although the prognosis for endovascular treatment was different between anterior circulation and posterior circulation, it was unexpected that there was not any clear correlation between vascular territory for FR in these studies [5, 25, 26]. We think it could be because the poor prognosis for stroke after ET depends not only on the occurrence of FR, but equally on other factors. Delay from missed diagnosis, technical difficulty, and the recanalization rate may be possible cause. Therefore, high baseline NIHSS score, older age, and longer delay have been identified as possible predictors for poor outcome after complete recanalization.

### 3.2. Imaging Markers

Owing to our research of recent articles about prediction of recanalization, it was found that some of the image markers can be used as the accurate predictors for poor prognosis after successful treatment. The recently published studies attempted to use some imaging criteria for selection of patients for mechanical thrombectomy to improve the odds of good outcomes.

#### 3.2.1. Large DWI-DWM Lesion

Tateishi and his team [27] found that large ischemic lesions in the deep white matter (DWM) on pretreatment diffusion-weighted MRI (DWI) might be a probable predictor for futile recanalization. The author defined large DWI-DWM lesion as a hyperintense lesion in the DWM on first DWI, located mainly between the anterior and posterior horns of the lateral ventricle. In 35 of 46 consecutive patients (76%) with complete recanalization, 20 patients after successful recanalization had a poor prognosis finally. Higher baseline NIHSS scores and older age could predict futile recanalization which was consistent with previous research; the study also found that a higher prevalence of large DWI-DWM lesions is associated with futile recanalization (45 versus 9%; *p* = 0.022). The positive predictive value for utile recanalization was nearly 90%. Patients with large preintervention DWI-DWM lesions may be poor candidates for endovascular therapy. However, the author also found that ASPECTS on CT and on DWI and initial ischemic lesion volume on DWI were not confirmed to have apparent correlation with futile recanalization which was inconsistent with other studies. It was probably due to small sample size and some of them did not use the new technique of endovascular therapy. Similarly, the usefulness of DWI-DWM needs to be confirmed.

#### 3.2.2. Leukoaraiosis

Leukoaraiosis (LA) is a radiological phenomenon that represents white matter lesions in the brain. Prior research indicated that hypoperfusion ischemia is probably the main cause for white matter abnormalities. The hypothesis is that structural and functional microvascular abnormalities in patients with LA have been supported by studies before. The brain tolerance from ischemic might be reduced when moderate-severe LA was diagnosed. It was unpromising to make the decision for recanalization procedures with patients with moderate-severe LA, because it often indicates poor microvascular cerebral reserve so that after vessels occlusion irreversible brain infarct rapidly develops [28, 29]. A recent study conducted by Gilberti and her teammates [24] was aimed at evaluating whether LA could predict futile recanalization in patients with anterior circulation LVO. 68 patients treated with endovascular therapy and achieving complete recanalization were included: 22 patients of them had a poor prognosis. By using of multivariable analysis, they found that moderate-severe LA was an independent predictor of FR (*p* = 0.01).

#### 3.2.3. ADC Quantification of Ischemic Lesions at Baseline MRI

In a small series [30], the researcher proved the prognostic value of baseline ADC quantification in patients with BAO undergoing EVT. 11 patients with BAO undergoing EVT were retrospectively investigated. They found that the lower values of minimum ADC at admission MRI are strongly correlated with higher scores in mRS at discharge (*p* = 0.009). And there was a negative correlation between minimum ADC and NIHSS at admission (*p* = 0.02), mRS at three months, and difference between pre- and posttreatment ischemic area (*p* = 0.026). Ischemic area and TICI grade were not significantly associated with clinical results. ADC quantification of ischemic lesions at baseline MRI seems to predict clinical outcome in patients with BAO undergoing EVT, more importantly than ischemic area or TICI grade.

#### 3.2.4. ASPECTS on CT Angiography Source Images

The recently published studies found that the appropriate selection of patients for mechanical thrombectomy upon imaging criteria helps improve prognosis. The Alberta Stroke Program Early CT Score (ASPECTS) [31] from initial noncontrast CT (NCCT) could predict FR which the pervious study mentioned [32, 33]. But the reliability of this score was low. Its usefulness for decisions about thrombolytic therapy has been debated, and a lower baseline ASPECTS score was one of the exclusion criteria for most recent trials of MT [34]. ASPECTS on CT angiography source images (CTA-SI-ASPECTS) was a more reliable indicator of outcome and final infarct volume in acute ischemic stroke [35, 36]. 110 patients with acute stroke from the FUN-TPA study registry [37] were included. All of the patients included had anterior circulation LVO and received reperfusion therapies. Kawiorski and his teammates attempted to assess whether the baseline of this score might help predict response to treatment and futile recanalization after reperfusion therapies reliably [25]. Total recanalization rate was 71%; 28% of cases were futile recanalization. Initial CTA-SI-ASPECTS was correlated with futile recanalization (OR 0.5; 95% CI 0.3–0.7); however NCCT-ASPECTS was not correlated with futile recanalization (OR 0.8; 95% CI 0.5–1.2). The score of CTA-SI-ASPECTS less than five was the optimum cut-off for prediction of futile recanalization (positive predictive value 86%; negative predictive value 77%; sensitivity 35%; specificity 97%). CTA-SI-ASPECTS is a reliable predictor for futile recanalization and could be used for treatment decisions for revascularization therapies.

#### 3.2.5. Collateral

A recent study [38] investigated whether the evaluation of collateral can be used as prediction for futile recanalization in acute ischemia stroke after recanalization. Collateral plays an important role in the pathophysiology of acute ischemic stroke and is identified as a conceivable predictor of FR. 135 anterior circulation stroke patients who received intravenous thrombolysis were retrospectively analyzed in this research. They finally put forward an equation using their collateral score (adjusting for baseline NIHSS, age, and recanalization) which emerged as a statistically significant prognostic biomarker for good prognosis (*p* < 0.033) among patients after completed recanalization, but not appropriate for nonrecanalized patients (*p* < 0.497). The results showed that collateral score was a reliable marker for prediction (*p* < 0.044). The poor collaterals predict poor prognosis despite successful recanalization and robust collaterals warrant consideration for recanalization therapy promoting the chance of better prognosis. Some other studies confirmed these conclusions in patients after ET.

#### 3.2.6. Combined Score on Multimodal Computed Tomography

Another study [23] aimed to assess the accuracy of parameters on multimodal computed tomography (CT) we mentioned before. The author also tried to use their combination for predicting futile recanalization after endovascular treatment. They retrospectively analyzed the data of a cohort of consecutive patients with stroke of anterior circulation. 57% of the patients among 150 patients included had futile recanalization. They accessed the predictive ability of ASPECTS on nonenhanced CT, CT angiography source images, CBF, CBV, mismatch CBV–CBF, and poor collaterals for futile recanalization. Among these parameters, ASPECTS on CT angiography source images ≤ 5, ASPECTS on CBV ≤ 6, and poor collaterals could predict futile recanalization independently after multivariate analyses. The combined score consisted of these parameters and could provide much more information: 57% of the patients with score 1, 89% with score 2, and 100% with score 3 had futile recanalization. Reclassification analyses indicated that the combined multimodal CT score predicted futile recanalization reliably. The prognostic value of this score needs more large studies to confirm.

Successful treatment of stroke patients especially with occluded vessel requires rapid and effective reperfusion therapies, which include mechanical thrombectomy and other endovascular treatment. One of the keys to success is the proper selection of patients in order to perform not only successful recanalization but also successful recovery. Treatment according to standards in current guideline was not enough. Futile recanalization was unexpected and not uncommon. Some of the indicators for futile recanalization in the past studies have been mentioned in this article which could be used for supplement for the clinician to make suitable decision. Further research is needed to understand the mechanism of futile recanalization and the correlation with cerebral blood flow regulation.
